# Psychometric properties of the disease-specific health-related quality of life instrument VascuQoL in a Swedish setting

**DOI:** 10.1186/1477-7525-10-45

**Published:** 2012-04-30

**Authors:** Joakim Nordanstig, Jan Karlsson, Monica Pettersson, Christine Wann-Hansson

**Affiliations:** 1 Department of Vascular Surgery and Institute of Medicine, Department of Molecular and Clinical Medicine, Sahlgrenska University Hospital, Bruna Stråket 11 B, 413 45, Gothenburg, Sweden; 2 Institute of Health and Care Sciences, Sahlgrenska University Hospital and Academy, Gothenburg, Sweden; 3 Centre for Health Care Sciences, Örebro University Hospital, Örebro, Sweden; 4 Department for Vascular Disease, Skåne University Hospital and Faculty of Health and Society, Malmö University, Malmö, Sweden

**Keywords:** Patient-reported outcome measures, Health-related quality of life, Peripheral arterial disease, Intermittent claudication, Psychometric validation

## Abstract

**Background:**

Traditional outcome measures in peripheral arterial disease (PAD) provide insufficient information regarding patient benefit. It has therefore been suggested to add patient-reported outcome measures. The main aim of this study was to validate the Swedish Vascular Quality of Life questionnaire (VascuQoL) version, a patient-reported PAD-specific health-related quality of life (HRQoL) instrument.

**Methods:**

Two-hundred PAD patients were consecutively recruited from two university hospitals. Out of the 200 subjects, 129 had intermittent claudication and 71 had critical limb ischemia. Mean age was 70 ± 9 y and 57% of the participants were male. All patients completed SF-36 and VascuQoL at the vascular outpatient clinic, when evaluated for invasive treatment. Risk factors and physiological parameters were registered. Construct validity was tested by correlation analysis versus SF-36 and was also assessed with multitrait/multi-item scaling analysis (MTMI). Sensitivity analysis regarding disease severity identification was performed. Reliability was assessed with Cronbach's alpha and responsiveness by standardized response mean (SRM) calculations.

**Results:**

Significant correlations were demonstrated between relevant subscales of VascuQoL and SF-36. MTMI showed acceptable construct validity, but some scaling-errors. VascuQoL significantly (p < 0.001) discriminated claudicants from critical limb ischemia patients. Cronbach's alpha was 0.94 and SRM 1.02 (sum score).

**Conclusions:**

The Swedish version of VascuQoL is valid and quantifies central aspects of HRQoL in PAD patients. Sensitivity analysis showed high ability to differentiate between disease severity and SRM illustrated excellent responsiveness. The relative abundance of items however makes use in the everyday clinical setting somewhat difficult.

## Background

Invasive treatment of peripheral arterial disease (PAD) improves limb-salvage in critical limb ischemia [1] and potentially improves functional status in intermittent claudication (pain in the legs during walking exercise, due to reduced blood flow caused by atherosclerosis). Traditionally used clinical outcome measures for evaluating the impact of disease as well as the effects of invasive procedures in PAD include amputation-free survival, walking capacity (e.g. treadmill testing), patency of revascularized segments, and physiological measurements such as the ankle-brachial index (ABI). With regard to patient benefit, these objective measures may have several limitations. Firstly, they do not provide sufficient information about the actual daily functional capacity of the PAD patient. Secondly, the degree of pain discomfort as experienced by the patient is not covered by traditional measures. And thirdly, they do not assess the impact of disease with respect to social and emotional consequences of living with PAD.

Therefore, it has been suggested that traditional measures should be supplemented with patient-reported outcome measures (PROMs) such as health-related quality of life (HRQoL) instruments [2]. Such measures can provide information of importance for clinical decision-making and assessment of results of different treatment strategies, both in clinical practice and research.

HRQoL may be analyzed using generic or disease-specific instruments. One well-known example of a generic instrument is the Medical Outcomes Study Short Form 36 (SF-36), which is the most widely used general health status measure. It is designed to provide assessments regarding generic health concepts, making it applicable for use in wider populations, irrespective of age, disease and treatment. Due to their general nature, generic instruments make comparisons possible between different populations and patients groups. On the other hand, generic instruments may be too insensitive to reveal smaller, but clinically important changes, because they do not focus on the specific problems experienced by PAD patients [3]. Moreover, these patients often are affected not only by leg ischemia but often also by several associated comorbidities that may negatively influence HRQoL after a successful vascular procedure. Therefore, the usage of disease-specific measures may be more appropriate as a pre- versus post intervention measure [4]. For PAD, such instruments are generally scarce and at present, no modern, formally validated disease-specific instrument is available for use in Sweden.

Vascular Quality of Life questionnaire (VascuQoL), a PAD-specific HRQoL instrument was developed at the Vascular Surgical Unit of King’s College Hospital in London [5]. The instrument has been translated to several languages [6], has been validated in the UK [7], and was for instance used in the BASIL trial [8]. Due to potential cultural and linguistic differences, most HRQoL experts opine that any HRQoL instrument should be formally validated before put to clinical use in a new country [9]. The main purpose of this study was therefore to evaluate the validity, reliability and responsiveness of the Swedish version of VascuQoL.

## Methods

### Study population

Two hundred subjects with established diagnosis of PAD were consecutively recruited from vascular centers at two university hospitals in Sweden. Out of the 200 subjects, 129 had intermittent claudication and 71 had critical limb ischemia. All patients independently completed the Swedish VascuQoL questionnaire and also the SF-36 instrument at the vascular outpatient clinic, when evaluated for invasive treatment. At one of the vascular centers, 127 of the participating patients also completed both questionnaires six months after a vascular intervention.

### Questionnaires

#### VascuQoL

VascuQoL aims at assessing quality of life over the entire spectrum of PAD, i.e. both in intermittent claudication and in critical limb ischemia. It consists of 25 items, subdivided into five domains: pain (four items), symptoms (four items), activities (eight items), social (two items), and emotional (seven items). Each question has a seven-point response scale. The responses are averaged to give an overall and a domain score ranging from one (worst HRQoL) to seven (best HRQoL).

VascuQoL was translated to Swedish and linguistically validated by the MAPI institute in France, specialized in linguistic validations of patient-reported outcomes instruments worldwide [6]. In close collaboration with the original developers of health outcomes instruments, the MAPI Institute has translated and linguistically validated more than 500 instruments into over 151 languages in a wide range of therapeutic areas.

#### SF-36

SF-36 is a well-established generic HRQoL questionnaire, validated among patients with PAD in Sweden [10]. It includes 36 items covering different aspects of HRQoL in eight different domains (PF = physical functioning; RP = role physical; BP = bodily pain; MH = mental health; RE = role emotional; SF = social functioning; VT = vitality and GH = general health). The response rates are ranging from yes or no, to a three-to-six response scale. Results are also presented in two summary measures, physical health summary (pcs) and mental health summary (mcs). Possible score ranges between 0–100 where 100 represent best possible HRQoL.

### Additional measurements

Ankle-brachial index (ABI), blood pressure at the ankle level divided by the blood pressure in the right forearm) was measured using a non-directional pen doppler and a standard blood pressure cuff. The highest value at each ankle level was recorded. Information regarding prospectively defined cardiovascular risk factors was collected from the medical records.

### Psychometric and statistical methods

#### Content validity

Five Swedish PAD experts subjectively evaluated face validity of VascuQoL independently, analyzing the different items in the translated Swedish version, with respect to relevance for peripheral arterial disease.

#### Construct validity

The latent construct of VascuQoL was tested by correlation analysis versus the different SF-36 subscales, using Pearson correlation coefficient. Multitrait/multi-item scaling analysis (MTMI) was also used to test scaling assumptions. This approach has been extensively used for the validation of HRQoL measures [11]. Ceiling-and floor effects were evaluated through histogram plots of normality.

#### Reliability

For reliability analysis, Cronbach's alpha was calculated for each subscale of VascuQoL. Cronbach's alpha is a measure of internal reliability, or consistency, i.e. how closely related a set of items is as a group. A Cronbach's alpha value of > 0.7 is generally considered acceptable for group data, while 0.9 is recommended for individual assessment [12].

#### Sensitivity and responsiveness

Sensitivity was evaluated by testing the ability of VascuQoL to discriminate between subjects with intermittent claudication and critical limb ischemia, using the non-parametric Mann–Whitney U test. Standardized response mean (SRM) was used for responsiveness testing and was calculated for each domain of VascuQoL, SF-36 and ABI (SRM = mean difference in score after vascular intervention as compared to baseline, divided by the standard deviation of the difference). Cohen's criteria [13] for interpreting effect sizes were applied (small = 0.2–0.5, moderate = 0.5–0.8, large > 0.8). Relative efficiency (ReE) was also calculated for relevant subscales of VascuQoL and corresponding SF-36 subscales using the formula ReE = (*t*1/*t*2)^2^, where *t*1 is the *t* value of a Students paired *t* test for the relevant VascuQoL subscale and *t*2 the relevant corresponding SF-36 subscale.

Statistical analysis was performed using SPSS version 19.0 (SPSS Inc. Chicago, IL, USA). The MTMI analysis was performed using MAP-R for Windows, version 1.0.

### Ethical considerations

Ethical approval was obtained from the Regional Ethical Review Boards at the University of Lund (No. 315/2008) and Gothenburg (No. 501/2009).

## Results

### Study population and missing data

Mean age was 70 ± 9 years and 57% of the subjects were male. About one-half (49%) of the subjects were previous or current smokers, 29% had diabetes and 43% had angina pectoris or a previous history of myocardial infarction (Table 1). Missing data was low (0–3%) over all for the 25 VascuQoL items, indicating that all questions are possible to interpret and answer for the responders. In the final dataset, 183 out of the 200 patients (92%) had complete HRQoL registrations and all patients had computable scales (using the half-scale method).

**Table 1:** **Demograhics and risk factors in patient population,*n*** **= 200**

**Demographics and risk factors**	
Age (mean + SD)	70 + 9 y
Gender (male/female; %)	57/43
Regulary smoking (current or in the last five years), %	49
Previous TIA or stroke, %	10
Diabetes, %	29
Hyperlipidemia, %	11
Angina pectoris/previous myocardial infarction, %	43
Chronic pulmonary disease, %	13
Kidney disease (s-creatinine >150 mmol/l), %	10
Ankle-brachial -index (mean + SD)	0.70 + 0.18

### Validity

#### Content validity

Regarding face validity, all five PAD experts agreed that VascuQoL covers central aspects of PAD disease. Questions were raised about the relevance of the seven-point response option and a reduction of possible response options was suggested.

#### Construct validity

Correlation analysis showed significant correlations between several SF-36 subscales and VascuQoL domain scores. The strongest correlations were noted between VascuQoL activity subscale and SF-36 physical subscale (PF) (r = 0, 69; p < 0.01) and between VascuQoL emotional subscale and SF-36 mental subscale (MH) (r = 0, 68, p < 0.01). Also, the VascuQoL pain subscale correlated strongly with SF-36 bodily pain, as did VascuQoL total score and SF-36 physical component summary, psc. This pattern was similar but augmented six months after vascular intervention and VascuQoL activity subscale correlated more strongly with PF (r = 0,86; p < 0.01). Stronger correlations were at that time also noted between VascuQoL pain subscale and BP (r = 0, 78; p < 0.01) and between the VascuQoL total score and SF-36 physical health summary (psc), (r = 0,79; p < 0.01), Table 2.

**Table 2:** Cross-sectional construct validity - correlations (Pearson) between VascuQoL and SF-36 scale scores

**VascuQoL**						
**SF-36**	**Total score**	**Pain**	**Activities**	**Symptoms**	**Emotional**	**Social**
PF	0.65**	0.52**	0.69**	0.54**	0.49**	0.61**
	n = 198	n = 200	n = 199	n = 200	n = 199	n = 199
	0.83**	0.74**	0.86**	0.72**	0.73**	0.73**
	*n = 127*	*n = 127*	*n = 127*	*n = 127*	*n = 127*	*n = 127*
RP	0.53**	0.47**	0.51**	0.48**	0.42**	0.48**
	n = 195	n = 197	n = 196	n = 197	n = 196	n = 196
	0.70**	0.62**	0.66**	0.62**	0.65**	0.69**
	*n = 126*	*n = 126*	*n = 126*	*n = 126*	*n = 126*	*n = 126*
BP	0.67**	0.62**	0.62**	0.57**	0.58**	0.49**
	n = 196	n = 198	n = 197	n = 197	n = 197	n = 197
	0.73**	0.78**	0.67**	0.69**	0.66**	0.57**
	*n = 127*	*n = 127*	*n = 127*	*n = 127*	*n = 127*	*n = 127*
GH	0.49**	0.36**	0.43**	0.38**	0.50**	0.44**
	n = 196	n = 198	n = 197	n = 198	n = 197	n = 197
	0.50**	0.41**	0.47**	0.45**	0.54**	0.40**
	*n = 127*	*n = 127*	*n = 127*	*n = 127*	*n = 127*	*n = 127*
VT	0.61**	0.49**	0.51**	0.55**	0.56**	0.49**
	n = 197	n = 199	n = 198	n = 199	n = 198	n = 198
	0.65**	0.57**	0.59**	0.60**	0.67**	0.53**
	*n = 127*	*n = 127*	*n = 127*	*n = 127*	*n = 127*	*n = 127*
SF	0.34**	0.21**	0.19**	0.29**	0.41**	0.43**
	n = 197	n = 199	n = 198	n = 199	n = 198	n = 198
	0.65**	0.51**	0.60**	0.60*	0.66**	0.66**
	*n = 127*	*n = 127*	*n = 127*	*n = 127*	*n = 127*	*n = 127*
RE	0.47**	0.37**	0.38**	0.41**	0.46**	0.41**
	n = 196	n = 198	n = 197	n = 198	n = 197	n = 197
	0.69**	0.60**	0.63**	0.60**	0.69**	0.72**
	*n = 126*	*n = 126*	*n = 126*	*n = 126*	*n = 126*	*n = 126*
MH	0.58**	0.40**	0.41**	0.49**	0.68**	0.49**
	n = 197	n = 199	n = 198	n = 199	n = 198	n = 198
	0.62**	0.52**	0.52**	0.54**	0.70**	0.58**
	*n = 127*	*n = 127*	*n = 127*	*n = 127*	*n = 127*	*n = 127*
PCS	0.59	0.53**	0.64**	0.50**	0.40**	0.52**
	n = 192	n = 194	n = 193	n = 194	n = 193	n = 193
	0.79**	0.74**	0.80**	0.71**	0.68**	0.66**
	*n = 126*	*n = 126*	*n = 126*	*n = 126*	*n = 126*	*n = 126*
MCS	0.49**	0.31**	0.28**	0.42**	0.60**	0.45**
	n = 192	n = 194	n = 193	n = 194	n = 193	n = 193
	0.57**	0.45**	0.46**	0.49**	0.65**	0.58**
	*n = 126*	*n = 126*	*n = 126*	*n = 126*	*n = 126*	*n = 126*
**ABI**	0.27**	0.15	0.22**	0.29**	0.24**	0.26**
	n = 143	n = 145	n = 144	n = 145	n = 144	n = 144
	*0.09*	*0.17*	*0.10*	*0.12*	*0.01*	*0.11*
	*n = 112*	*n = 112*	*n = 112*	*n = 112*	*n = 112*	*n = 112*

Correlations at six-months follow-up in italics (n = 121).

** Correlations are significant at the 0.01 level, (two-tailed test).

*PF* = physical functioning; *RP* = role physical; *BP* = bodily pain; *GH* = general health; *VT* = vitality; *SF* = social functioning; *RE* = role emotional; *MH* = mental health; *PCS* = physical health summary; *MCS* = mental health summary and *ABI* = ankle-brachial-index.

MTMI analysis was conducted to test the scaling assumptions of VascuQoL and the results are summarized in Table 3. Item-scale correlations indicated satisfactory convergent validity for the subscales pain, social and emotional, (i e correlations exceeded the minimum desired level, r = 0.4). The activity subscale performed generally well although one of eight item-scale correlations (item 9, “During the past two weeks, the distance I can walk has improved....”) was too low. The symptom subscale showed a relatively poor convergent validity.

**Table 3:** Summary of results of multitrait/multi-item scaling tests of the Swedish VascuQoL questionaire

**Multitrait/multi-item scaling tests**					
	**Item-scale convergent validity**		**Item-scale discriminant validity**		**Scaling fulfillment**
	**Range of r**	**Criterion 1**	**Range of r**	**Criterion 2**	
**Scale**	**Item-scale correlations^a^**	**% of item scale correlations ≥ 0.40^b^**	**Correlation with other scales^c^**	**Number of items higher**	**Number of items that meet both criteria 1 and 2^e^**
**Activity**	0.25–0.71	88	0.04–0.70	7/8	7/8
**Pain**	0.51–0.66	100	0.38–0.66	2/4	2/4
**Emotional**	0.59–0.76	100	0.42–0.64	5/7	5/7
**Social**	0.55–0.55	100	0.48–0.70	0/2	0/2
**Symptom**	0.3–0.55	50	0.29–0.63	0/4	0/4

^a^Pearson correlations between items and hypothesized scale (corrected for overlap).

^b^Number of item-scale correlations that meet the minimum standard for convergent validity (r ≥ 0.40)/total numbers of correlations.

^c^Pearson correlations between items and competing scales.

^d^Correlations higher between items and hypothesized scale in comparison with all other scales.

^e^Items in each scale that meet criteria for both item-scale convergent (Criterion1) and discriminant (Criterion 2) validity.

Regarding discriminant validity, the subscales activity and emotional performed well, mainly correlating higher with their own scale than with the other subscales. The subscales pain, social and symptom were shown to have relatively poor discriminant validity (Table 3). VascuQoL correlated poorly to the baseline ABI values (Table 2). The change in ABI did not correlate to change in VascuQoL sum or domain scores.

#### Reliability

For the VascuQoL total score, Cronbach's alpha was 0.94 and for the subscales alpha values were acceptable (alpha ≥ 0.7), except for the symptom subscale that had a somewhat weak alpha value (0.64), Table 4.

**Table 4:** Crohnbach’s alpha values for the different VascuQoL domains, with respect to disease severity and after vascular intervention

**Scale**	**Total**	**Intermittent claudication**	**Critical limb ischemia**	**Six months after vascular intervention**
**Activity**	0.87 (n = 189)	0.86 (n = 126)	0.78 (n = 63)	0.94 (n = 124)
**Pain**	0.77 (n = 195)	0.74 (n = 126)	0.78 (n = 69)	0.89 (n = 127)
**Emotional**	0.88 (n = 192)	0.85 (n = 127)	0.89 (n = 65)	0.94 (n = 127)
**Social**	0.70 (n = 196)	0.67 (n = 127)	0.68 (n = 69)	0.84 (n = 127)
**Symptom**	0.64 (n = 194)	0.56 (n = 127)	0.61 (n = 60)	0.73 (n = 126)
**Total**	0.94 (n = 183)	0.93 (n = 123)	0.92 (n = 60)	0.97 (n = 123)

#### Floor- and ceiling effects

Proportions of subjects scoring at the lowest possible scale level (floor effect) were marginal for all subscales (≤2.5%). Ceiling effects (proportion of subjects scoring at the highest possible scale level) were also negligible, although 6.5% of the subjects scored at the maximum scale level of the social subscale. Floor-and ceiling effects for the different VascuQoL subscales with respect to disease severity are given in Table 5.

**Table 5:** Sensitivity analysis and floor-ceiling effects of VascuQoL with respect to disease severity, (Mann Whitney U)

**Scale**	**Intermittent claudication (IC), mean ± SD n = 127**	**Critical limb ischemia (CLI), mean ± SD n = 71**	**p-value**	Floor* (IC), %	**Ceiling (IC), %**	Floor* (CLI), %	**Ceiling (CLI), %**
**Activity**	3.50 ± 1.12	2.44 ± 0.88	p < 0.001	0	0	4.2	0
**Pain**	3.69 ± 1.18	2.61 ± 1,10	p < 0.001	0.8	0	8.5	0
**Emotional**	4.57 ± 1,29	3.35 ± 1,48	p < 0.001	0	0.8	2.8	1.4
**Social**	4.34 ± 1.60	3.17 ± 1,63	p < 0.001	0	7.1	5.6	5.6
**Symptom**	4.46 ± 1.09	2.97 ± 1,23	p < 0.001	0	0	5.6	0
**Total**	4.05 ± 1.03	2.87 ± 1.00	p < 0.001	0	0	1.4	0

Higher scores indicates better health status.

*Proportion of subjects scoring at the lowest possible scale level.

Proportion of subjects scoring at the highest possible scale level.

#### Sensitivity and responsiveness

**Figure 1:**
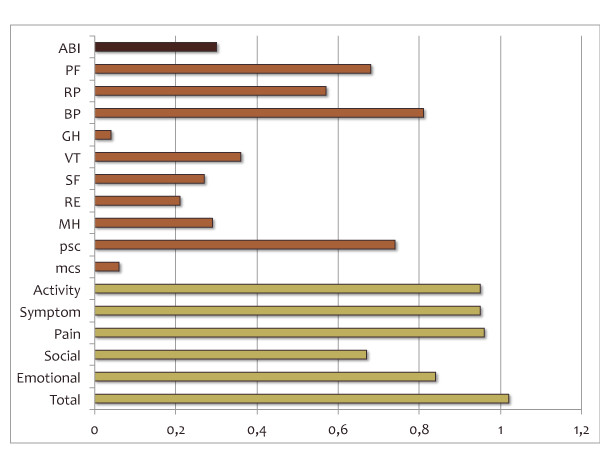
**Standardized response mean (SRM) after vascular intervention; for ABI (ankle-brachial index) and the different VascuQoL and SF-36 subscales.***ABI* = ankle-brachial index; *PF* = physical functioning; *RP* = role physical; *BP* = bodily pain; *GH* = general health; *VT* = vitality; *SF* = social functioning; *RE* = role emotional *MH* = mental health; *PCS* = physical health summary and *MCS* = mental health summary. Cohen’s criteria for interpreting effect size: 0,0–0,2 = trivial; 0,2–0,5 = small; 0,5–0,8 = moderate; >0,8 = large.

VascuQoL was shown to discriminate significantly between subjects with intermittent claudication and critical limb ischemia (Table 5). Standardized response mean (SRM) for total and domain scores of VascuQoL, calculated for the 123 patients that completed the HRQoL instruments also six months after a vascular intervention, are given in Figure 1. SRM for VascuQoL total score was 1,02, indicating a large effect. All but one (social scale) of the VascuQoL subscales demonstrated a SRM value over 0.8, which, according to applied criteria [13], indicate excellent responsiveness. In comparison with SRM for the different SF-36 scores and ABI, VascuQoL performed better (Figure 1). Relative efficiency (ReE) of the VascuQoL activity subscale vs. the SF-36 physical functioning subscale was 2,2, for VascuQoL pain subscale vs. SF-36 bodily pain subscale ReE =1,5; for VascuQoL emotional subscale vs. SF-36 role emotional subscale ReE = 15,3 and for VascuQoL social subscale vs. SF-36 social functioning subscale ReE = 8,1.

## Discussion

The main finding in this study is that VascuQoL, a PAD-specific HRQoL assessment questionnaire, is valid, reliable and responsive, thereby adequately reflecting central aspects of HRQoL among patients living with PAD in Sweden. The instrument showed a high ability to differentiate between patients with intermittent claudication and patients with critical limb ischemia, indicating a high sensitivity to quantify HRQoL in this patient group. It also seems to be a reliable instrument, as judged by high values of Cronbach's alpha for the sum and most subscale scores. Moreover, responsiveness seems to be excellent as illustrated by our SRM calculations and the instrument would therefore be ideal as an additional outcome measure after vascular surgical intervention. VascuQoL has been validated in the UK before [7], but the Swedish culture and treatment options may differ, why it is important that the instrument is now also formally validated from a Swedish culture and context [9].

In comparison with the analysis made by the original VascuQoL developers [5], we found a similar pattern with regard to construct validity and responsiveness. The VascuQoL domains activity and pain correlated strongly with PF and BP respectively. We noticed a somewhat stronger correlation (r = 0.68) between VascuQoL emotional and MH, possibly explained by our larger patient sample. Regarding responsiveness, setting the limit for acceptable results at a SRM >0.5 [13], all domain scores of VascuQoL performed well, whereas only three (PF, RP and BP) out of eight domains of SF-36 were responsive. Similar findings were also made in the Dutch version of VascuQoL by de Vries et al [3]. Clinical indicators such as ABI correlated poorly to HRQoL scores, as have been demonstrated by other authors [5,7,14]. To summarize these findings, our present data confirm that VascuQoL is a valid instrument for measurements of outcomes after vascular interventions.

Minimal important difference (MID) of a change in HRQoL is ideally determined using patient-based and clinical anchors [15]. Our data set did not allow for such an approach as we did not use transition questions. Moreover, we found that the ABI values correlated poorly to HRQoL, making their use as clinical anchors problematic [16]. However, as described by Norman et al [17] the threshold of discrimination for changes in HRQoL appears to be around half a standard deviation (SD). Using this definition, minimal important difference (MID) for VascuQoL total score in our sample would be 0,58. Applying this on our data set, 72% of our subjects reached a MID after vascular intervention. Setting the limit for MID at 0,2 SD would result in a MID for VascuQoL total score of 0,23 and 76% of the subjects reaching MID.

There are a few concerns about some parts of the scale constructs, with possible implications for the validity of the instrument. Multitrait-multi-item scaling tests indicated scaling errors in all domains, although most were of minor importance. Weak item-scale convergent and discriminant validity were shown for the symptom scale. However, since this scale contains items on symptoms that can cause deterioration in HRQoL (so-called “causal indicators”) the construct should be regarded as a clinimetric scale [18]. Traditional psychometric scaling analysis is based on the assumption that all items are “effect indicators” reflecting the same latent construct, thus leading to high correlation structure and high internal consistency. Causal indicators on the other hand are usually more heterogeneous, leading to weaker correlation structure and lower internal consistency. Evaluation of clinimetric scales should be based on content validity and clinical usefulness rather than internal consistency [18]. The satisfactory responsiveness demonstrated in the present and other studies [5,7] indicates that the symptom scale is of clinical importance for measuring effects of treatment.

Item-scale convergent validity was satisfactory for the pain, emotional, social and activity domains, with one exception. One item (item 9) in the activity domain correlated weakly, indicating that this item may be removed without loss of information. This may be explained by the somewhat illogical construction of that item, i e one cannot expect that the patients have improved their walking capacity at baseline. Item-scale discriminant validity was generally weaker, especially for the social and pain domains. The social domain consists of only two items which both correlated higher with other domains. The social domain may perhaps be improved by adding one or more additional items. Items in the pain domain were somewhat unspecific with moderate correlations with several scales.

One possible disadvantage, which was also commented by the expert group in our study, is the seven-point response option. For example, the 6-point response scale used in the SF-36 version 1 for measuring VT and MH was changed in version 2 to a 5-point scale. Elimination of one response choice simplified the form without loss of information [19]. This suggests that further development and tests of VascuQoL should include a reduction of the seven-point response scale.

For the vascular surgery community, it is important to integrate patient-reported outcome measures in daily practice. Such measurements offer important additional information about the vascular patient that could be used for the evaluation of different invasive treatment modalities and possibly also as an evaluation tool for proper selection of patients to offer invasive treatment [20,21]. Moreover, they are important additional outcome variables in clinical research. Of special importance is the evaluation of different treatment options with seemingly similar short- and long-term objective outcome (e.g. regarding amputation-free survival; mortality etc.). In such cases it is obviously very important to offer the patient the procedure that results in less HRQoL reduction, caused by the surgical procedure per se. This is for instance the case sometimes when comparing endovascular and open vascular procedures. HRQoL measures ideally could offer such important information in daily practice. But at the same time, these instruments ought to be easy to fill out and contain a limited amount of items, to be really practical to use in the broader everyday clinical setting.

Limitations of our validation strategy includes that we, for resource reasons, did not perform test-retest reliability assessment. Moreover, due to the somewhat limited study sample, our dataset did not allow for formal factor analysis that could demonstrate how well the hypothesized factors explain the observed data.

## Conclusions

In conclusion, our data shows that the Swedish VascuQoL version is a valid HRQoL instrument in PAD, confirming previously observed results regarding the original English version [5]. Although there are a few improvements in the scale construct to be made, the performance in our study was generally acceptable-to-good. Drawbacks of VascuQoL concern mainly the relatively abundance of questions and the seven-point response scale, making broad use in the everyday clinical setting somewhat difficult. A short version of the instrument may be possible and more practical, and we will continue our work in that direction.

## Competing interests

The authors declare that they have no competing interests.

## Authors’ contributions

JN and C W-H designed the study and collected the data. JN performed most statistical analysis, and JN and C W-H participated in interpretation of data and writing of the manuscript. JK performed the MTMI statistical analysis, took an integral part in analysing and interpretation of data and also in writing of the manuscript. MP participated in analysing and interpretation of data and writing of the manuscript. All authors read and approved the final manuscript.
